# What trauma patients need: the European dilemma

**DOI:** 10.1007/s00068-022-02014-w

**Published:** 2022-07-07

**Authors:** Falco Hietbrink, Shahin Mohseni, Diego Mariani, Päl Aksel Naess, Cristina Rey-Valcárcel, Alan Biloslavo, Gary A. Bass, Susan I. Brundage, Henrique Alexandrino, Ruben Peralta, Luke P. H. Leenen, Tina Gaarder, the Visceral Trauma Section of the European Society of Trauma, Emergency Surgery

**Affiliations:** 1 Department of Surgery, University Medical Center Utrecht, Heidelberglaan 100, 3584 CX Utrecht, The Netherlands; 2 Division of Trauma and Emergency Surgery, Department of Surgery, Orebro University Hospital and School of Medical Sciences, Orebro University, 702 81 Orebro, Sweden; 3 Department of General Surgery, ASST Ovest Milanese, Milan, Italy; 4 Department of Traumatology, Oslo University Hospital Ulleval, Oslo, Norway; 5 Department of Surgery, Hospital General Universitario Gregorio Marañón, Madrid, Spain; 6 General Surgery Department, Cattinara University Hospital, Trieste, Italy; 7 Division of Traumatology, Surgical Critical Care and Emergency Surgery, University of Pennsylvania, Philadelphia, USA; 8 Department of Surgery, R Adams Cowley Shock Trauma Center, Baltimore, USA; 9 Department of Surgery, Coimbra University Hospital Centre, Coimbra, Portugal; 10 Department of Surgery, Trauma Surgery, Hamad General Hospital, Doha, Qatar; 11 Department of Surgery, Universidad Nacional Pedro Henriquez Urena, Santo Domingo, Dominican Republic; 12 Hamad Injury Prevention Program, Hamad Trauma Center, Hamad General Hospital, Doha, Qatar; 13 Department of Surgery, University Medical Center Utrecht, Utrecht, The Netherlands

**Keywords:** Trauma systems, Traumasurgery, Critical care, Surgical skills, Education in trauma

## Abstract

There is a need for implementation and maturation of an inclusive trauma system in every country in Europe, with patient centered care by dedicated surgeons. This process should be initiated by physicians and medical societies, based on the best available evidence, and supported and subsequently funded by the government and healthcare authorities. A systematic approach to organizing all aspects of trauma will result in health gain in terms of quality of care provided, higher survival rates, better functional outcomes and quality of life. In addition, it will provide reliable data for both research, quality improvement and prevention programs. Severely injured patients need surgeons with broad technical and non-technical competencies to provide holistic, inclusive and compassionate care. Here we describe the philosophy of the surgical approach and define the necessary skills for trauma, both surgical and other, to improve outcome of severely injured patients. As surgery is an essential part of trauma care, surgeons play an important role for the optimal treatment of trauma patients throughout and after their hospital stay, including the intensive care unit (ICU). However, in most European countries, it might not be obvious to either the general public, patients or even the physicians that the surgeon must assume this responsibility in the ICU to optimize outcomes. The aim of this paper is to define key elements in terms of trauma systems, trauma-specific surgical skills and active critical care involvement, to organize and optimize trauma care in Europe.

## Introduction

Despite differences in geography, human lifestyle, political climate and economic environment in Europe, trauma is still the leading cause of death for people under the age of 40, although mortality rates vary widely between countries [1]. The ongoing effort to improve trauma care in each country is often lead by forerunners within the field of traumatology, which resulted in trauma care to evolve in different ways within Europe. In some countries, dedicated processes have resulted in updated trauma systems being implemented, in others less so. To provide some common denominators and standardization, the different generic elements of a trauma system and individual skill set of physicians involved need to be described, compared, and investigated.

## The need for trauma systems

It has been postulated that the implementation of an inclusive trauma system results in reduced mortality for severely injured patients [2–5]. A mature trauma system yields improved quality of life [6, 7] and a cost reduction for every saved life [8, 9]. Finally, trauma systems with adequate registration processes lead to robust information concerning aspects to focus on for prevention [10, 11]. Key trauma system characteristics include mainly political and logistical elements, such as: the assignment of a lead agency responsible for maintaining the system, the assurance that the system provides a continuum of services, rules for triage systems for patient allocation including bypass of non-designated trauma hospitals, criteria for secondary transfers to trauma centers, criteria for in-hospital infrastructure and competence, and continuous system-wide evaluation for quality improvement [12]. There are many options in how to design such a system in Europe [13] including who should be the responsible physicians and what their tasks should be. Although direct comparison of even adjusted outcomes between institutions and countries have limited value, the differences in practice between countries would provide the opportunity to learn from each other [14]. Regardless of the differences, trauma systems with adequate data registration can play an essential role in prevention programs.

### Geographic and demographic circumstances will have an important impact on the organizational structure of a trauma system

A low density populated area with mountains, hard weather conditions and long transportation distances will require a different organization of both the pre-hospital services, as well as the trauma level designation compared to a more densely populated area where shorter transport times are possible. This results in differences in thresholds for pre-hospital response times, as well as the threshold to deploy physicians in addition to paramedics in the field [15]. Prehospital services also vary widely depending on resources. Whereas in systems with short transportation times it might be possible to present all severely injured patients primarily to a Major Trauma Centers (MTC), this might not be achievable as transportation time increases. In the latter case, it might be more desirable to stabilize the patient in a lower-level trauma receiving hospital (LTH) before transportation to an MTC for definitive care [16]. This has significant consequences for the design of the system, resources needed, and the necessary skill set of the surgeons involved throughout the whole system.

### Political and societal factors are important for the implementation of trauma systems

Several countries in Europe still have a long way to go to develop robust nationwide systems necessary to optimize trauma care. No country has been able to implement changes on a national level unless the government was involved in the trauma system development and maintenance. Although the “power” balance in decisions regarding trauma care between government and medical (societies) will differ between countries, government participation is possible by formulating legislation, quality control or financial support. In some countries, additional funding is part of incentives to improve trauma care. In others, the threat to lose emergency or trauma functions has been an efficient inducement to fill the necessary requirements.

In most countries, a change toward a trauma system was initiated by a few (trauma) surgeons or medical societies, based on scientific reports or guidelines, presented to, and supported by, the government. In these instances, investments in personnel, teaching and training and nationwide protocols supported by the government and healthcare authorities has led to significant improvement in outcomes [17–22]. Further introduction, implementation and maintenance within an established system requires active support by the government. In some countries with a mature inclusive trauma system, but without dedicated government support, the primary delivery of multiply injured patients (ISS > 16) to a MTC stagnated to around 50% [23–27]. In contrast, some of the most well developed and organized trauma systems in the world are the result of physician-initiated, but with far reaching governmental support and financial steering [17, 28, 29]. Unfortunately, in some European countries there is a lack of an organized trauma system, which leaves potential to improve care for the injured in terms of reduced mortality and functional recovery.

## Trauma is a surgical disease

Most European countries with an established trauma system have chosen a form of surgical trauma care with either general or orthopedic surgeons in the lead for the severely injured patients. In comparison, in the USA the general surgeon is in the lead and during last decades combining trauma surgery with acute care surgery and/or intensive care medicine. This format has become more widespread, especially at designated Level-1 Trauma Centers throughout the country [30]. In contrast, in Europe most surgeons do not practice intensive care medicine (anymore) [31] and other models have evolved.

In several European countries, the orthopedic surgeon is the lead of trauma care. These systems have evolved from the “original trauma surgeon (“der Unfallchirurg”)”, based on the fact that > 80% of the surgical procedures performed after trauma is fracture surgery. In systems with orthopedic surgeons as trauma leaders, it might be challenging to organize and perform timely lifesaving (visceral: cervical, thoracic or abdominal) procedures or surgery. Although from a ‘surgical procedure perspective’ this might be a fulfilling model [32], it does require a large amount of organizational work to keep (preventable) mortality limited. For optimal care and clinical outcomes, surgical involvement is essential during each step including resuscitation, damage control surgery, intensive care support, definitive surgery to recovery phase in the ward, rehabilitation clinic and outpatient department.

On the other hand, there are two types of health care systems with the general surgeon as trauma lead. In most of the countries in Europe who embraced the US model, the general surgeon functions as an acute care surgeon who treats the torso injuries of trauma patients and surgical emergencies in non-trauma patients, leaving skeletal injuries to orthopedic surgeons. The challenge in these countries is to keep sufficient focus and dedication to trauma and provide attractive surgery to those who are dedicated to trauma in systems with less frequent exposure on operative management in trauma.

Finally, some countries still keep the initial model of trauma surgery (“Unfallchirurg”) in place, who treats both the torso injuries and extremity injuries [33]. With increasing non-operative management of visceral trauma, this model has the challenge to maintain competency of surgeons with these injuries and their operative management.

There might be some regions with a non-surgical specialty in the lead of the severely injured patient, most of the time due to the vacuum of surgical expertise. However, it might be challenging to incorporate a broad surgical skillset into the decision-making, both on a holistic level as well as on a more detailed level during primary and secondary surgical procedures. This will require a tremendous amount of organizational and communication skills, is threatened by fragmented care and might not be a universal model for trauma. In most cases, the non-surgical specialist might take the lead during a specific phase of the patient journey, but will not be the captain on the patients’ ship from harbor to harbor.

## Surgical knowledge and skills

The acute and complex character of severely injured patients is what makes trauma surgery both appealing and challenging to many surgeons [34], requiring broad knowledge of physiology and anatomy, as well as communication, leadership and organizational skills, in addition to all aspects of lifesaving surgery. The outcome of severely injured patients is determined by the whole chain of care provided, which is highly dependent on multidisciplinary and inter-disciplinary cooperation [35].

### Non-technical skills are important

Leadership, communication, organizational skills and logistical management are among the most important assets of a trauma surgeon. Almost by definition, severely injured patients require a multidisciplinary team approach [36, 37]. In hectic and pressurized situations, the system should be pre-programmed with a robust mindset for surgical decision-making and procedure execution [38]. Routines should be maintained by simulated team-practice on a regular basis [29, 39–41]. As quoted, ‘it is not to rise to the occasion, but to decent to your level of training’. This training and standardization should not be confined only to the management in the emergency department (ED), it should include every step from admission until discharge to rehabilitation and thus including multiple different teams throughout the journey. Ideally, the trauma surgeon guides the patient during the whole chain of care.

### Indication and timing of interventions is key to favorable clinical outcomes

It is well known that severely injured patients require timely care [42]. Trauma surgery was even coined as the example of time-sensitive care, but minimal time between injury and treatment and minimizing time per treatment given is not the only aspect to this matter. This whole concept encompasses indications for treatment (surgical and/or non-surgical), the extent of the surgical procedure (including the details during tissue handling and manipulation by the surgeon), the use of abbreviated surgery, the timing of the treatment and when needed a stepwise approach, or the omission of (operative) interventions when physiology or immunology does not allow for it. The decision not to operate is as surgical as the decision to operate. Consequently, the injured patient requires a surgeon with knowledge of both physiology and surgical procedures in trauma to tailor the most optimal management. Familiarity with physiology, immunology, resuscitation and critical care support is of paramount importance. Furthermore, it is essential the surgeon knowns his/her own operative skillset and the impact (burden) of different choices on the physiology of the patient. To obtain this expertise, trauma must be a significant part of the trauma surgeons’ education and dedicated daily activity. As described by others, damage control surgery is not just fast elective surgery [43, 44], it requires the surgeon to build a mindset, and apply surgical techniques and decision-making that differ from other (more elective) surgical specialties [45]. To learn this knowledge, it can be taught during residency and fellowships, with additional training and courses for maintaining learned skills when deemed necessary, depending on exposure. The goal should be confidence in making the decision to perform lifesaving procedures and damage control surgery of truncal and junctional injuries.

The skills needed, regardless of a background in either general or orthopedic surgery, to treat severely injured patients can be learned from courses provided internationally, such as the ATLS^®^/ETC^®^ and DSTC^®^/DSATC^®^, ATOM course [46]. The skills taught in these courses are minimum requirements to take care of a severely injured patient, and modular add-ons might be needed in different settings. For instance in some geographical regions, it might be desirable or needed to add lifesaving neurosurgical procedures to this package. Stabilization of extremity injuries by means of splinting or external fixation and including shunting or repair of vascular injuries are other competencies of added value during the initial phase of resuscitation. The need and requirements of skills (medical and non-medical) should be evaluated per system.

### Quality control cycles

Frequent analysis of the injured patient journey within every part of a trauma system is mandatory to optimize the logistics and facilities within a center and to learn as a team. Regular feedback to all involved in the trauma care is important for dissemination of knowledge [47]. This might be done during mortality and morbidity meetings and may even have more impact in distinct trauma pathway debriefings. This ensures involvement of all parties and places focus on the injured patient as a different entity [48]. These meetings can also function to discuss or implement new strategies and techniques. In most centers, many medical specialties are involved in the care of the severely injured patient. Frequently, identified potential for improvement relates to poor communication, need for updated protocols, need for education and regular trauma team CRM (crew resource management) simulations.

The recipe to obtain sufficient competences as a trauma surgeon could be formulated as follows: modular training and maintaining the basic skills set x repeatedly analyzing patient journeys x updating current knowledge/needs.

### A trauma surgeon works in a dynamic environment that demands constant adaptation

The aging population in many European countries poses new challenges. Fragile bones, anticoagulants, atherosclerotic arteries and severe comorbidities frequently form the background to which the injuries are to be dealt with [49, 50]. In parallel, new techniques, devices and procedures become available at a higher pace than ever before. Currently, endovascular treatment in a hybrid suite for operative, endovascular and non-operative management of the bleeding patient is spreading rapidly [51, 52]. Reports on the downsides of these new modalities and the cost of infrastructural changes can help determine its limitations and thereby placing it correctly in the arsenal of possibilities for the injured patient. It is the task of the trauma surgeons to determine the risks, potential correct indication and timing of these new techniques.

## The trauma patient in the critical care unit

One of the founding fathers of trauma surgery in Europe, Martin Allgöwer, stated several decades ago that the surgeons were abandoning the intensive care unit and that this exodus would have significant impact on their patients’ care [31]. Currently, the ICUs in Europe are mainly staffed by intensivists with a background in anesthesiology, internal medicine, neurology or cardiology [53]. The combination of surgery and intensive care seems either undesirable or unachievable for most surgeons in Europe. We want to emphasize that surgical involvement during the critical care period of severely injured patients is essential to optimize clinical outcomes as surgical mindset, skills and knowledge tends to be complementary to the intensivist’s knowledge [54].

Knowledge of physiology, pathophysiology and immunology is the foundation on which intensive care medicine is build. For the surgeon, to be of added value in the ICU, understanding of these aspects is mandatory [55–60]. This requires the combination of a physician with a background as a generalist in combination with specific expertise regarding injured patients. Knowledge concerning intensive care is barely transferred in most surgical residency programs in Europe [61]. Focus is on technical skills, performing in theatre and communication skills for adequate interaction with patients. In many surgical curricula in Europe, education in physiology boils down to only a short period of intensive care medicine during the residency, if at all. Sufficient experience is not gained to master this field. Therefore, when pursuing a career in trauma surgery, the common residency programs should have increased focus on intensive care medicine, or fellowships and scholarships should be offered. Nevertheless, it has been stated that often, surgeons do not have the time to master intensive care medicine in addition to meeting the demands of operative practice [62]. This is amplified by the current European work hour regulations, already limiting time to master operative procedures. This lack of fulltime surgical involvement and interest in the ICUs in Europe, contributed to the development of closed format ICU’s with limited surgical involvement, treating trauma patients.

The introduction of the closed format intensive care, with appointed intensivists, has been demonstrated to reduce morbidity and mortality when all patients are analyzed [63]. The main hypothesis behind this success, is that it is better to have one person orchestrating the total care, rather than “breaking” the patient up into organ systems, calling for an integrated approach [62]. This same integrated approach holds true for the chain of care provided for the severely injured patient and thus these two visions are corresponding and should be aligned [64].

Nevertheless, it is important to understand the differences in point of view between surgeons and intensivists because the added value of surgical expertise is direly needed during the critical care phase of trauma patients. Regardless of the intensive care format used (closed, open, mixed) and whether a surgeon or intensivist is in charge, surgical involvement is essential as ‘surgical critical care is based on surgical decision making’ [65, 66]. Close collaboration is key and requires trauma surgeons to build the necessary competence to be complimentary to the expertise offered by the intensivists.

## Building stronger trauma care in Europe

Trauma systems are necessary to optimize trauma care and are still lacking partly or entirely in many European countries. Successful trauma systems have the same generic elements, which include dedicated physicians with governmental involvement and support (Table 1). The specifics of the system will depend on geographical and demographical structure and the only way to determine the systems’ performance is through adequate and complete data from (national) trauma registries. Although existing trauma systems in Europe have evolved with differences at every level, the injured patient is always in need of the same standard of care everywhere and thus always requires a surgeon who possesses a skill set to diagnose and perform lifesaving procedures. Hence, the trauma surgeon should be (recognized as) a specialist who has knowledge of both physiology, injuries and life-saving surgical procedures. Trauma is a surgical disease in need of surgical decision-making, whether operative or not. As such, surgical leadership is paramount in the care of the severely injured and should be trained, including communication and teamwork in addition to the list of technical skills learned in advanced trauma courses. The trauma surgeons should be the glue that holds all elements together for the injured patient, also during the intensive care treatment of these patients. This requires expertise, patient and process ownership and decisional authority [5]. For this to happen, communication is essential between surgeons and intensivists. The model chosen can differ between countries, with either a surgical intensivist staffing or surgical co-ownership with dedicated intensivists, but in the end, teamwork is essential to do “the greatest good to the greatest number” per trauma system [67].

**Table 1:** Generic elements to take on trauma as a disease

Subject	Highlights
Trauma systems	Design of a system
Geography and demography	Determine dedicated logistics (prehospital/triage)
Political and societal factors	Government support and financial steering
Background of primary specialty	General, orthopaedic or combined surgery as core specialty determine organization and team build up
Surgical knowledge and skills	Technical and non-technical skills
Leadership and organization	Teamwork, communication and coordination is key, everybody in the team should be trained in trauma
Indication and timing	Knowledge of (patho)physiology and the impact of surgical procedures is necessary
Essential skillset required	Minimum requirements for lifesaving procedures and stabilization of extremity injuries
Feedback by patient journeys	Multidisciplinary analysis of the whole trauma chain to identify areas for further improvement
Adaptive mindset	Changing populations and changing techniques require constant adjustment of strategies
Critical care knowledge	Critical care is an essential step in trauma care
The ICU is among the most critical periods after trauma	Surgical involvement is key: critical care decision-making is based on surgical decision-making
Knowledge of (patho)physiology, and immunology required	To be of added value as a surgeon in the critical care period adequate expertise is necessary
The trauma patient needs an integrated approach	Taking care of the trauma patient from the beginning to the end may improve outcome
